# A systematic review of goal attainment scaling implementation practices by caregivers in randomized controlled trials

**DOI:** 10.1186/s41687-024-00716-w

**Published:** 2024-03-26

**Authors:** Kulpreet Cheema, Taylor Dunn, Chere Chapman, Kenneth Rockwood, Susan E. Howlett, Gunes Sevinc

**Affiliations:** 1 Ardea Outcomes, Halifax, NS Canada; 2 Department of Pharmacology, Dalhousie University, Halifax, NS Canada; 3 Division of Geriatric Medicine, Dalhousie University, Halifax, NS Canada; 4 Geriatric Medicine Research Unit, Nova Scotia Health Authority, Halifax, NS Canada; 5 Neuroscience and Mental Health Institute, Faculty of Medicine and Dentistry, University of Alberta, Edmonton, AB Canada

**Keywords:** GAS, Goal attainment scaling, Systematic review, Caregivers

## Abstract

**Background:**

Goal attainment scaling (GAS), an established individualized, patient-centred outcome measure, is used to capture the patient’s voice. Although first introduced ~60 years ago, there are few published guidelines for implementing GAS, and almost none for its use when caregivers GAS is implemented with caregiver input. We conducted a systematic review of studies that implemented GAS with caregiver input; and examined variations in GAS implementation, analysis, and reporting.

**Methods:**

Literature was retrieved from Medline, Embase, Cochrane, PsycInfo and CINAHL databases. We included randomized controlled trials (published between 1968 and November 2022) that used GAS as an outcome measure and involved caregiver input during goal setting.

**Results:**

Of the 2610 studies imported for screening, 21 met the inclusion criteria. Most studies employed GAS as a primary outcome. The majority (76%) had children as study participants. The most common disorders represented were cerebral palsy, developmental disorders, and dementia/Alzheimer’s disease. The traditional five-point GAS scale, with levels from −2 to +2, was most often implemented, with −1 level typically being the baseline. However, most studies omitted essential GAS details from their reports including the number of goals set, number of attainment levels and whether any training was given to GAS facilitators.

**Conclusions:**

GAS with caregiver input has been used in a limited number of randomized controlled trials, primarily in pediatric patients and adults with dementia. There is a variability in GAS implementation and many crucial details related to the specifics of GAS implementation are omitted from reports, which may limit reproducibility. Here we propose catalog that may be utilized when reporting research results pertaining to GAS with caregivers to enhance the application of this patient-centered outcome measure.

**Supplementary Information:**

The online version contains supplementary material available at 10.1186/s41687-024-00716-w.

## Background

Goal attainment scaling (GAS), an established individualized, patient-centered outcome measure, has been applied across multiple disciplines to capture the patient voice [1–5]. This outcome measure provides both qualitative and quantitative information on progress towards goal attainment after an intervention or treatment and allows assessment of clinically meaningful change that is unique to each patient. The individualized nature of GAS makes it suitable for use in disorders with heterogeneous symptoms and disease progression [2], especially where generic outcome measures fail to achieve the required sensitivity and specificity [6].

GAS was first introduced by Kiresuk and Sherman in 1968 to evaluate outcomes in a mental health setting [7]. Since then, it has been successfully adapted for use in many other domains, including stroke rehabilitation [8, 9], drug trials [2], back pain [10], rehabilitation [3], communication disorders [11], and in other older adults with dementia [1], and complex needs [12]. There is growing evidence that GAS is sensitive to change and can capture clinically meaningful changes that often are ignored or are not elicited by standardized tests [6]. For instance, in an investigation of the feasibility, validity and responsiveness of GAS in long-term care, researchers reported that among several other measures, GAS was the most responsive measure, with an effect size of 1.29 and a relative efficiency of 53.7 [13]. Several other reports have also emphasized its responsiveness in capturing treatment effects [14–19].

The goal setting process starts with an interview between the patient (and/or caregiver) and an interviewer to identify those goals that are most important to each individual patient. For each unique goal, typically a baseline level at −1 and four other attainment levels are set, ranging from +2 (much better than the goal, best-expected outcome) to −2 (worst than the goal, worst expected outcome) [7]. A post-intervention assessment is performed where the goal rater (e.g. the patient or caregiver and/or clinician) describes the level of attainment achieved for each goal. Then for each participant a GAS score, called the T-score, is calculated based on the formula proposed by Kiresuk and Sherman [7]:

$${\text{T}} = {50 + (10\sum {({{\text{w}}_i}{{\text{x}}_i})})}/\surd\,((1 - \rho)\sum {{\text{w}}_i^2} + \rho\,{(\sum {{\text{w}}{{\text{i}}_i}})^2})$$

w_*i*_ = weight assigned to the ith goal

x_*i*_ = numerical value of the goal attainment achieved (between −2 and +2)

ρ = expected correlation of the goal scales.

The T-score allows an expression of goal attainment in multiple goal scales in a single score for each patient. The formula assumes scales to be normally distributed, and usually, a mean T-score of 50 indicates that all goals were attained.

GAS goals are often identified and set by the patient to reflect their own personal goals of treatment with input from clinicians or other trained personnel [3]. However, in many circumstances, patients may be unable or minimally able to participate in goal setting. Patient input may be inadequate to set treatment goals and/or to assess attainment levels in studies of interventions in children or in people who live with cognitive impairment. Under these circumstances, caregivers, in partnership with the patient and/or clinician, can help with individualized goal setting, and evaluation of goal attainment after an intervention. In this context, caregivers are defined as those individuals that provide support and are close to the patients so that they are attuned to the patient’s challenges and needs. They provide input to goal scale development and assessment when the patients lack cognitive (i.e. older adults with dementia) and/or communicative capacity (children with severe developmental delay).

Several reviews have addressed aspects of GAS including its reliability [20], validity [21], and utility in specific disorders [1–23]. In addition, Logan et al. [4] recently reviewed GAS implementation practices when it is used as an outcome measure in randomized controlled trials. They reported that GAS implemented by patients provides data on individualized outcomes in a wide range of disorders [4]. However, much less is known about GAS implementation when caregivers take part in setting goals and assessment of attainment levels. The objectives of this review are to determine the most common GAS implementation practices with caregiver input in the context of clinical trials, and assess variations in the implementation, analysis, and reporting.

## Methods

### Protocol and registration

We conducted a systematic review search according to the PRISMA guidelines [24]. The search protocol was registered in PROSPERO in October 2021.

### Databases and searches

**Fig. 1:**
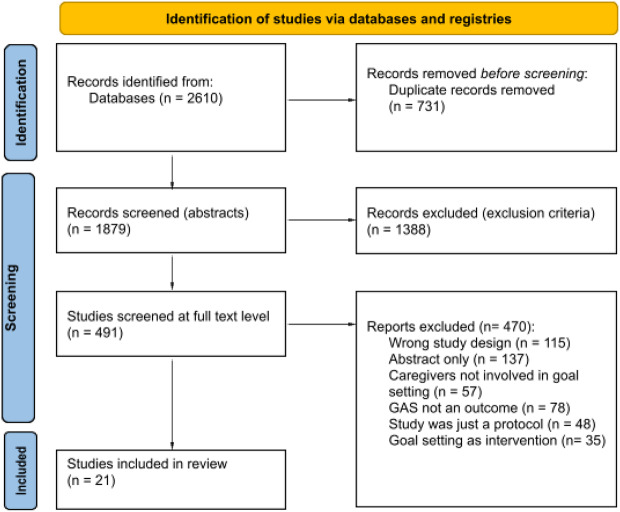
The PRISMA 2020 flow diagram for new systematic reviews. This includes searches of databases and registries only. *From* Page MJ, McKenzie JE, Bossuyt PM, Boutron I, Hoffmann TC, Mulrow CD, et al. The PRISMA 2020 statement: an updated guideline for reporting systematic reviews. BMJ 2021;372:n71. doi: 10.1136/bmj.n71. For more information, visit: http://www.prisma-statement.org/

We searched Medline, Embase, Cochrane, PsycInfo and CINAHL databases for literature on GAS. The included studies needed were from 1968 (the year when GAS was first introduced; [7]) to Nov 27, 2022. Searches of the following keywords were performed:

Randomized controlled trials, family care*, carer*, caregiver*, prox*, parent*, goal set*, goal plan*, goal attain*, goal achiev*, care* goal*. The full search strategy is included as a supplementary materials file (Supplementary File 1). The PRISMA flowchart is illustrated in Fig. 1.

The reference lists of the articles that were included in the full-text screening stage were also consulted to find additional articles eligible for this study. The inclusion criteria were:

1. Randomized controlled studies
2. A caregiver (or a proxy or a parent) was involved in the goal setting process, with or without the input of patients through an interview with a GAS rater (or clinician).
3. The goals are personalized for each subject
4. Published in English
5. Published after 1968.

Studies were excluded if they mentioned the concept of goal attainment but did not use any scaling procedure to evaluate goal achievement, or when goal setting was used as an intervention and not as outcome measure. Additionally, any reports that were simply conference abstracts or narrative reviews were excluded.

### Data extraction and quality assessment

Three researchers teamed up to screen and review the articles, of whom one (KC) screened all the abstracts and full texts. To ensure consistency of the abstract selection process by a single screener, 25% of the abstracts were screened by a second reviewer. In case of disagreement, a third reviewer (SH) was consulted to resolve the conflicts. This additional step was used to increase the confidence in having a single screener. The third reviewer (SH) was also consulted on the inclusion criteria at the full-text stage. Covidence software (Covidence.org) was used to perform the screening and data extraction. A standard data extraction form was created in the Covidence program.

We extracted information on study characteristics (number of sites, sample size, intervention details), and on risk of bias for randomized controlled trials (allocation sequence use, allocation sequence concealment, blinding of the participants and personnel, blinding of outcome assessment, completion rate of outcome assessments, reporting of all outcome assessments), GAS implementation details (number of goals set, number of levels set, personnel performing goal setting and attainment, quality assurance details, use of goal menu, weighting of goals, goal calibration) and GAS analysis details (types of treatment effects, effect sizes reported, statistical tests used). The full list of extracted variables is included in Supplementary File 2.

### Analysis

A descriptive and narrative synthesis of the implementation practices of goal attainment scaling was performed. This included synthesizing information on the most common approaches to goal setting, interview process, use of goal menus and different approaches to GAS score calculation.

We also reported and summarized effect size values as Cohen’s *d*. For the studies that did not report Cohen’s *d*, we calculated Cohen’s *d* where possible with the following formula:

$${\text{Cohen's}}\,d = {\text{M}}_{1}-{\text{M}}_{2}/{\text{pooled}}\,{\text{SD}}$$

where M = mean, SD = standard deviation.

The pooled standard deviation was calculated with the following formula:

$${\text{Pooled}}\,{\text{SD}} = \surd\, (({{\rm n}_1} - 1){\text{S}}{{\text{D}}_{\text{1}}}^2 + ({{\rm n}_2} - 1){\text{S}}{{\text{D}}_{\text{2}}}^2/({\rm n}_1 + {\rm n}_2 - 2)$$

n = sample size; SD = standard deviation

In cases of multiple effect sizes (i.e. multiple treatment groups or follow-up times), only the effect sizes for the highest dose and the final endpoint were reported. Two studies did not have sufficient information to calculate Cohen’s *d* [25, 26].

## Results

### Search results

The search resulted in 623, 964, 425, 129 and 469 abstracts from Embase, Cochrane, Medline, PsychInfo and CINAHL databases, respectively. Duplicate records (N = 731) were removed. The resulting articles (N = 1879) were screened at the abstract and title level. This excluded 1388 articles, leaving 491 for full-text review. The reasons for exclusion at the full-text review were the following: incorrect study design; abstract only; caregivers were not involved in goal setting; GAS was not an outcome measure; the work was simply a study protocol; or GAS was used as an intervention (Fig. 1). Based on this screening a further 470 studies were excluded, leaving 21 studies that were included in this review.

### Study characteristics

**Fig. 2:**
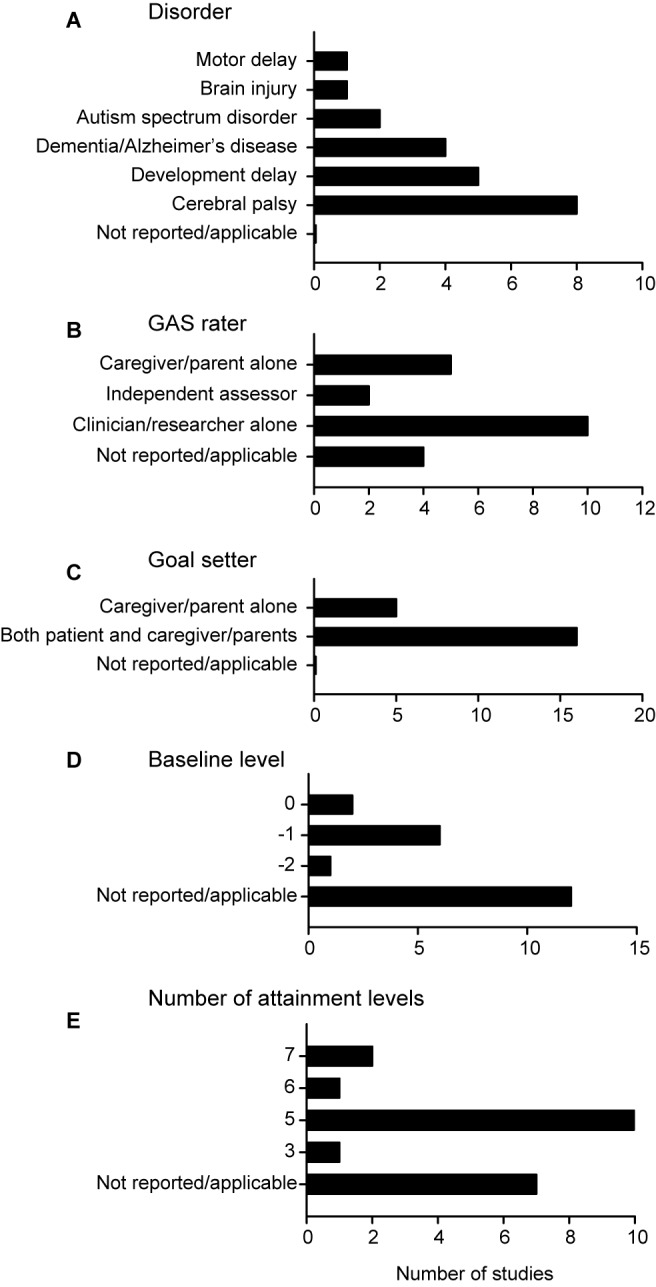
Characteristics of the GAS studies reviewed. **A** Most studies reviewed considered children with cerebral palsy or developmental delay. **B**, **C** The GAS raters were most often the clinician or researcher alone, while patients and caregivers together were most likely to set goals. **D**, **E** The most common baseline level was −1, and most studies used five attainment levels. Bar graphs depict the number of studies with each characteristic

Most of the studies were conducted in Australia, followed by the US and Canada (Table 1). The most common disorders in the review were cerebral palsy (38%), developmental delay (23%) and dementia/Alzheimer’s disease (19%); other disorders were less common (Table 1; Fig. 2A). The total sample sizes varied from 20 to 1533, with a median sample of 41 (The sample sizes from Rockwood [27, 28] were excluded from this calculation as they are from the same study). Sixteen studies (76%) had children as the study participants. The rest were adults. Eleven studies (52%) had GAS as a primary outcome, with 8 studies using the Canadian Occupational Performance Measure (COPM) as an additional patient reported outcome measure used in conjunction with GAS.

**Table 1:** Study and participant characteristics

Reference	Country (number of sites)	Participant characteristics	Sample size (intervention group(s))	Sample size (control group)	Range, mean age (years)	Intervention	GAS utility (primary or secondary outcome)
Armstrong et al. [29]	Australia (1)	Children with Developmental Delays	32	36	1.5–3 years, 2.4 years^a^	Use of LeAP playground in a community centre by children with developmental delays	Secondary
Cusick et al. [30]	Australia (1)	Children with spastic hemiplegic cerebral palsy	20	21	2–7 years, 3.9 years	Botulinum toxin A injections	Primary
Cusick et al. [31]	Australia (1)	Children with spastic hemiplegic cerebral palsy	21	21	2–8 years, 3.9 years	Botulinum toxin A injections	Secondary
DeJoode et al. [32]	Netherlands (7)	Adults with acquired brain injury	21	13	18–75 years, 40.8 years	Use of personal digital assistance as a cognitive aid	Primary
Dimitrova et al. [33]	Canada, Hungary, Philippines, Poland, Russia, South Korea, Thailand, Turkey, and the United States (40)	Children with upper limb spasticity	75 for 6 U/kg	79	2–17 years, 7.9 years^a^	Onabotulinumtoxin-A injection plus OT sessions	Secondary
			78 for 3 U/kg				
Hoare et al. [25]	Australia (1)	Children with congenital spastic unilateral Cerebral Palsy	17	17	1.5–6 years, 3 years	Botulinum toxin A injections	Secondary
Huang et al. [34]	Taiwan (1)	Children with motor delays	ROC-Sit: 15	11	1–3 years, 1.85 years	Ride-on car training	Secondary
			ROC- Stand: 12				
Hwang et al. [25]	Taiwan (8)	Children with or at risk for Developmental Delay	19	16	0.42–2.5 years, 1.45 years	Routine based early intervention	Primary
Leroi et al. [35]	UK (1)	Adults with Parkinson’s disease with dementia	11	13	Ranges not reported, 75.6 years	Administration of memantine 20 mg daily	Primary
Lowe et al. [36]	Australia (1)	Children with hemiplegic Cerebral Palsy	21	21	2–8 years, 4 years	Botulinum toxin A injections	Secondary
Lowe et al. [37]	Australia (3)	Children with Cerebral Palsy	42	40	2–8 years, 4 years	Botulinum toxin A injections	Secondary
McKean et al. [38]	Australia (1)	Children with speech disorder	10	10	3–6 years, 4.22 years	Family-centred practice	Secondary
McMorran et al. [26]	UK (1)	Children with Diplegic Cerebral palsy	30	32	Ranges not reported, 14.5 years	Surgery	Primary
Mills et al. [39]	Australia (1)	Children with Autism	16	18	4–12 years, 7.4 years^a^	School-based sensory activity schedule	Secondary
Olesch et al. [40]	Australia (1)	Children with hemiplegic cerebral palsy	11	11	1.5 to 5 years, 3.67 years	Botulinum toxin A injections	Primary
Rockwood et al. [18]	Canada (10)	Adults with mild to moderate Alzheimer’s disease	53	56	51–94 years, 77.5 years	Galantamine medication	Primary
Rockwood et al. [27]^b^	Canada (10)	Adults with mild to moderate Alzheimer’s disease	53	56	51–94 years, 77.5 years	Galantamine medication	Primary
Rockwood et al. [28]^b^	Canada (10)	Adults with mild to moderate Alzheimer’s disease	53	56	51–94 years, 77.5 years	Galantamine medication	Primary
Schaaf et al. [41]	United States (1)	Children with Autism spectrum disorder	17	14	4–8 years, 5.98 years^a^	Manualized intervention for sensory difficulties	Primary
Schasfoort et al. [42]	Netherlands (7)	Children with spastic cerebral palsy	41	24	4–12 years, 7.33 years	BoNT-A injection	Secondary
Tilton et al. [43]	United States (1)	Children with dynamic foot equinus	79 for both groups	76	2–17 years, no mean age reported	AbobotulinumtoxinA injection	Primary

^a^Mean age calculated by authors

^b^Total sample sizes for these two studies were not included in the final calculation as they are from the same study

### Risk of bias

Risk of bias was assessed using Cochrane’s risk assessment tool (version 1) and included the following criteria: allocation sequence use, allocation sequence concealment, blinding of the participant and personnel, blinding of outcome assessment, completion rate of outcome assessments, and reporting of all outcome assessments. Most studies utilized a generated allocation sequence in a sufficient manner (72%) and concealed it appropriately to prevent selection bias (57%). However, the participants and personnel were blinded to the intervention in only a few studies (14%), and the outcome assessment was conducted in a blinded manner to prevent performance bias and detection bias (19%). On the other hand, the majority of studies reported the completion rate of outcome assessment to prevent attrition bias (76%) and included all outcome assessments in their reports to prevent reporting bias (76%). The results of the risk assessment are visualized using robvis [44] and are included in Supplementary File 3.

### Application of GAS used by caregivers in clinical trials

**Fig. 3:**
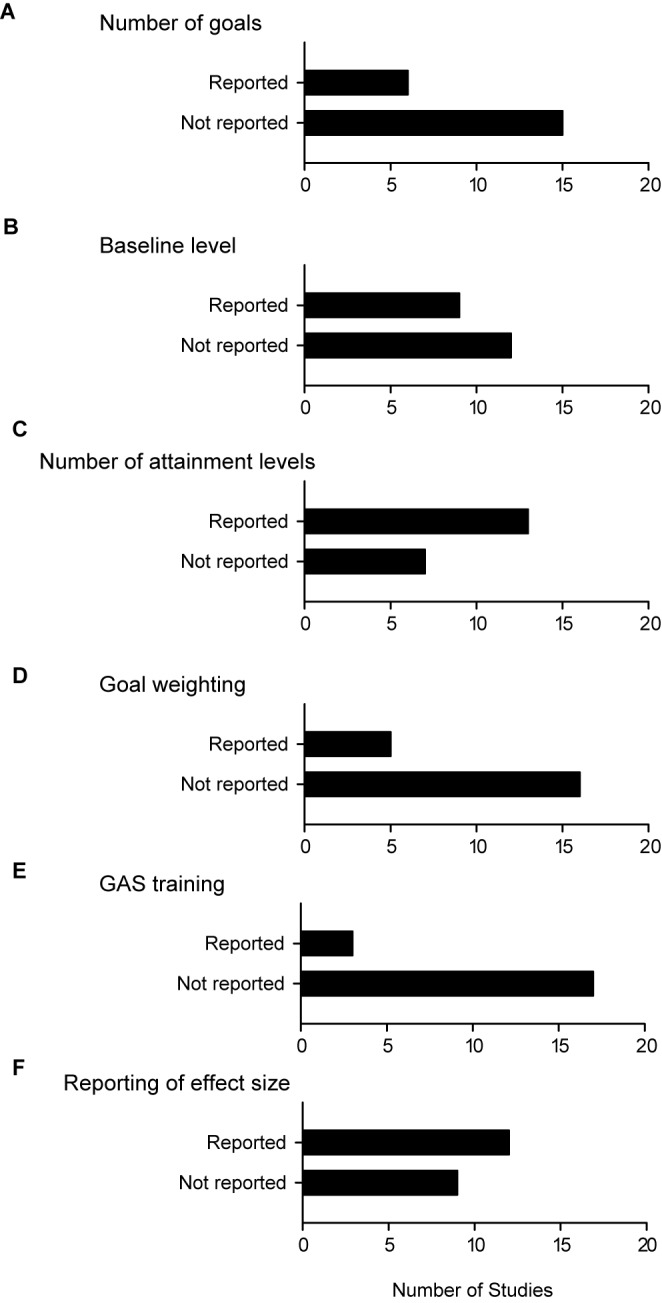
The number of studies that reported or did not report specific details used in GAS analysis. **A**–**F** Studies that used GAS did not routinely report many aspects of their GAS methodology and analysis. Bar graphs depict the number of studies with each characteristic that was either reported or not reported in the study reviewed. The sample size in each panel was 21

Several different approaches were used in employing GAS with caregiver input during goal setting (Figs. 2 and 3). Most studies (76%) reported that both the patient and the caregiver set goals collaboratively (76% of studies; Fig. 2C). In the remaining studies, only the caregiver was involved in goal setting. While caregiver input was utilized in setting goals, they were not always included in the assessment of attainment levels. In many cases the clinicians and/or researchers were the most likely to be involved in such assessment (e.g. GAS raters), followed by caregivers (Fig. 2B). Two studies noted that independent raters scored goal attainment (Fig. 2C). Most studies reported the use of −1 as the baseline, with 0 level being the next most used baseline (Fig. 2D). The traditional five-point GAS scale from −2 to +2 was most often implemented, with 10 studies (48%) using the classic 5 attainment levels (Fig. 2E). Two studies reported the use of a 7-point GAS scale ranging from −3 to +3; one study used a 6-point scale (from −2 to +3) (Fig. 2E).

Most studies did not report the number of goals set. Six studies (29%) specified the total number of goals set (Fig. 3A). The mean number of total goals set across all studies was 287 (median number of goals = 300.5; range = 30 to 516). The range of goals or mean/median number of goals set was rarely reported, with 3 studies reporting the mean and 1 study reporting the median number of goals set for each patient group. The mean number of goals per participant ranged from 2.2 to 3.7 (median number = 3 goals per participant). Under-reporting of the baseline level was also common, with 12 studies not reporting this information (Fig. 3B). Most studies (n = 14) reported the number of GAS attainment levels used (Fig. 3C). Five studies weighted the goals, and all were based on how important the goals were to the patient and/or caregivers (Fig. 3D).

Very few studies reported whether any training was given to the personnel conducting GAS, with only 3 out of the 21 studies (15%) reviewed indicating that GAS raters were trained (Fig. 3E). One study (5%) reported that GAS raters completed a 4-h training program [45] while another (5%) reported that raters completed an 8-h training program [9]. None of the studies reviewed stated that they used a formal goal menu or goal inventory during the goal setting process. The occupational therapy-based patient-reported outcome measure (COPM), was collected along with GAS in 8 studies. There was a mix of approaches with these measures, where some studies used GAS and COPM separately, while others used these measures in a complementary fashion. In this latter case, the goal categories in the COPM were used to help patients and caregivers with goal setting.

### GAS analysis

**Fig. 4:**
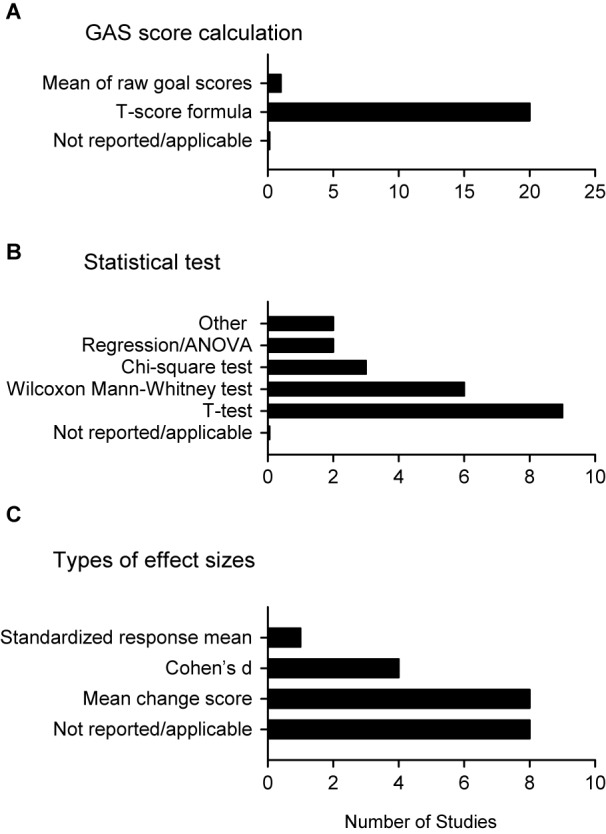
Analysis techniques used in the GAS studies reviewed. **A** All but one study calculated the GAS score with the traditional T-score formula. **B** Different statistical tests of significance were used, with the Student’s or unequal variance *t*-test being the most common. **C** Effect sizes were measured as the mean change score, Cohen’s *d* or the standardized response mean. Bar graphs depict the number of studies with each analytic technique. Where the sample size was greater than 21 (panel B), more than one analysis technique was used

Figure 4 illustrates the approaches used for GAS analysis. *T*-tests were generally used to analyze between-group treatment effects, with a Student’s *t*-test or an unequal variance *t*-test being the most common. This was followed by non-parametric tests, with the Wilcoxon Mann-Whitney and chi-square tests most often employed. Other statistical tests included regression analysis, analysis of variance and paired-sample *t*-tests (Fig. 4A, B).

All studies used the T-score formula originally proposed by Kiresuk & Sherman [23] to summarize the GAS scores. One study reported the mean of raw goal scores and divided participants in terms of those who responded to treatment and those who did not (Fig. 4A). Studies generally reported some measure of effect size (Fig. 3F), including mean change scores, standardized response means, and Cohen’s *d* (Fig. 4C). The most common effect size measure reported was mean change scores, with 8 out of 21 studies (39%) reporting this measure. We also summarized Cohen’s *d* values from all the studies included in this review to investigate the responsiveness of GAS. We found that Cohen’s *d* values ranged from −0.015 to 2.56 with a median effect size of 0.52. Most studies had medium to high effect sizes (e.g. effects sizes between 0.5 and 0.8), with the 4 studies showing small effect sizes (e.g. effects sizes between 0.1 and 0.4) [45].

## Discussion

Here we systematically investigated GAS implementation practices with caregiver input during goal setting in randomized controlled trials; and observed that GAS was employed in a limited number of randomized controlled trials, primarily in pediatric patients and adults with dementia. We also found out that the implementation and reporting of GAS implementation practices was inconsistent and often incomplete, which may affect assessments of GAS validity and may also hinder replication efforts.

Common practices for GAS were identified across the 21 studies. Both patients and caregivers were consulted for goal setting, with few studies including only the caregivers. The inclusion of caregivers along with the patient may help preserve the patient-centered nature of GAS, while allowing a caregiver to contribute to the treatment plan. While patient and/or caregiver input was utilized in setting goals, the clinicians and/or researchers were the most likely to be involved in the assessment of goal attainment, followed by caregivers, and independent assessors. There are multiple ways to include caregivers in the GAS process (only during goal setting, only during the assessment of goal attainment, or during both; with or without patient involvement) and best practices for caregiver involvement in GAS are yet to be developed.

The results of this review suggest that within the context of GAS, caregiver input is typically utilized not as proxy, but to complement patient input and, in line with the occasional appearance of term ‘proxy’ in the identified studies (only in [31, 40]), caregiver input is used as a substitute for patient input only when it is necessary to do so (i.e. patients with severe cognitive impairment and/or very young children). Yet, this warrants an examination of the association between patient and proxy (caregiver-as-proxy) responses. Researcher examining the accuracy of proxy reports indicated a differentiation between subjective and objective domains; and that the proxies had higher accuracy in objective domains such mobility, self-care, and activities, as opposed to more objective domains such as pain and emotional state, or other psychosocial domains [46, 47]. Other researchers examining accuracy of proxy-report indicated that they may be a reasonable alternative when patient self-report cannot be obtained and when group mean scores are averaged across individuals [48]. Yet, the researchers suggested that the proxy report should be interpreted more cautiously [49–51], especially when used to assess meaningful change at the individual level [48]. Additional research that explores potential divergence between patient and caregiver priorities during goal setting, and the impact of caregiver involvement on specific goal domains could be illuminating.

For the majority of studies, the quality assessment indicated a lower risk for selection, attrition, and reporting bias, with a higher risk for performance and detection biases. While a considerable number of studies reported medium to large effect sizes, the high risk in performance and detection bias and absence of blinding might have an influence on reported medium-to-high effect sizes. Future studies might benefit from minimizing such risk in the design execution, analysis, and reporting of randomised trials.

The recommendations for implementing GAS proposed by Kiresuk and Sherman [7] were followed by most researchers/clinicians including the use of a five-point scale, a value of −1 for the baseline and the use of the GAS T-score formula. The most common statistical test used to compare between-group treatment effects was a *t*-test. These details are critical for the use of GAS as an outcome measure and this suggests that, in general, these practices are being followed by most researchers. As the naming T-score may wrongly give the impression of truly standardized interval scale, application of quality appraisal criteria during goal scale development (e.g. [52, 53]), and testing for statistical assumptions is recommended (see [3] for a review).

In addition to documenting how GAS with caregiver input is deployed, we draw to attention the inconsistent and incomplete reporting of many details with respect to the GAS implementation. These include the failure to report the number of goals set, number of attainment levels set, and whether any training was given to GAS facilitators. Reporting these details would not only help in the assessment of the validity of the measure, but also will help in the replication of GAS by different investigators, across multiple disorders and in different contexts.

Similarly, information on the type of GAS training could also help to evaluate the quality of goal setting in studies. Three studies reported the type of training provided to GAS personnel [18, 30, 43]. Even in these studies, there were few details provided about the training, with two studies reporting the duration of the training, which ranged from 4 to 8 h. The description of the training also was vague. Cusick et al. [30] reported a training programme that included “GAS development, administration and scoring; reading, modelling, in-situ practice, feedback on administration and practice scales,” while Tilton et al. [43] noted that “Injectors were trained in applying GAS methodology and set SMART (Specific, Measurable, Achievable, Relevant, and Time-Bound) goals through series of workshops.” None of these articles referenced the use of any previous studies or guides on GAS training, despite published procedures for GAS rater training [54] and for writing SMART rehabilitation goals [55]. In addition, reviews and guides that describe how to implement GAS and goal setting across in different contexts [3, 55–59] and conditions such as dementia [60] could have been referenced as well. However, most of these guides are related to rehabilitation work, which might have inhibited investigators from other fields from consulting this literature.

Most studies with caregivers had children as the patient population. This is not surprising considering the critical role of caregivers in populations who require advocacy and help. This is important, as GAS has been recommended to improve the transparency and tuning of rehabilitation goals with parents during a child’s rehabilitation process [61]. Our review highlights the relevance of this measure to children as well. However, we have not examined whether the nature of the relationship within different patient-caregiver dyads (i.e. children and their parents, residential care workers or other paid care providers as caregivers), or patient population (i.e. children versus older adults) impacts GAS implementation. Future research the impact of this relationship on the type and number of goals set, assessment of attainment levels or other GAS-related criterion would be of interest.

Our review identified a relatively small number of clinical trials that used caregiver input to deploy GAS when compared to results of a recent review that examined the use of GAS in clinical trials in general [4]. This suggests that caregivers are used less frequently than the patients themselves, primarily in pediatric patients and adults with dementia. While all studies reviewed here had caregivers involved in goal setting (i.e. caregiver GAS), two studies had an additional GAS measurement performed by a clinician. In Rockwood et al. [18], the end-point standardized response mean was 0.22 for caregiver GAS and 0.38 for clinician GAS. In Lowe et al. [36], Cohen’s *d* was 2.56 for caregivers and 3.32 for clinicians. In both instances, the effect sizes between the clinician and caregiver GAS were close, although GAS attainment was rated higher by the clinician than by the caregiver. While these authors did not discuss any reasons for this difference, it is possible that this might be due to the clinician’s experience with goal attainment. Clinicians have access to other patients in the group, so their assessment might have been made in comparison to other patients. On the other hand, higher ratings might be due to the clinicians having an altered sense of a treatment effect with patient/caregiver perspectives providing a more realistic view of treatment effects, or due to the lack of unblinding. There is a need for more studies to compare caregiver and clinician perspectives to try to identify the reasons for these discrepancies.

Interestingly, no study reported using a specific goal inventory or goal menu to assist in goal setting. In some of the studies the categories in the COPM were used to complimentary fashion to help patients and caregivers with goal setting. Goal-menus or inventories have been used in goal setting in various disorders including hemophilia [62], neurogenic bladder [63], elder mistreatment [64, 65] and dementia [66] to aid in goal setting. The goal inventory can be prepared with input from patients [67], disease experts [66], and/or both [63], with special consideration given to the conceptual model of the disease and/or the product’s proposed mechanism of action. This may enhance detection of clinically meaningful results [68]. The inventory includes a list of common challenges that can be mapped to various domains and are realistically achievable following an intervention. This makes the goal setting process easier; and helps in standardizing goals, which might be of interest to regulators, as it conforms more with most standardized measures. Furthermore, analyzing the frequency with which specific goals occur or cluster by domain can potentially help elucidate the processes by which a drug leads to the desired pharmacological effects. On the other hand, when used rigidly, it might reduce sensitivity to novel treatment effects, especially early in the course of treatments being developed [69].

As many essential details of GAS implementation do not find their way into current reports, the reproducibility of GAS may be limited. A standardized approach for consistent reporting of GAS is one remedy. Therefore, in addition to using a quality appraisal criterion during the implementation of GAS (see [53] for an example of comprehensive quality appraisal criteria in rehabilitation), we propose a catalog (Table 2) to assist researchers and clinicians in reporting the important details of GAS. The catalog lists potential items to consider when reporting GAS implementation details and is divided into two categories: GAS administration and GAS analysis, with items identified as either suggested or optional. The suggested information includes critical details to be reported related to GAS application and analysis to facilitate study replication. For example, reporting the mean change score, sample size and standard deviation of GAS scores is suggested but calculating Cohen’s *d* is listed as optional as Cohen’s *d* can be calculated from the mean and standard deviation scores.

**Table 2:** Catalog of potential items to include when reporting GAS implementation details

Activity	GAS assessment	Potential GAS methods reported	Attribute reported **Yes/No/NA**^b^
Goal setting	Personnel involved in goal setting	Patient	
		Clinician	
		Caregiver	
		Other	
	Personnel involved in assessing goal attainment	Patient	
		Clinician	
		Caregiver	
		Other (e.g. independent rater)	
	Number of goals set	Mean number of goals in each group	
		Range of goals	
		Time spent setting goals	
	Weighting of goals	By importance	
		By difficulty	
		Other weighting criteria, specify	
	Quality assurance	Number of GAS levels set	
		Baseline level specified	
		Blinding to other test scores	
		^a^Use of goal menu or inventory	
		Goal quality assessment performed	
	Training of GAS interviewers/raters	Name of training program	
		Brief description of training	
		Duration of training	
	Number of GAS interviews completed	Total number of interviews	
		Number of interviews by group (if multiple groups)	
Analysis of GAS data	Calculation of GAS score	Raw score	
		T-score (T-score and standard deviation for each group)	
	Treatment effect size	Mean change scores (within and between groups)	
		Standard deviation of baseline and change scores	
		^a^Cohen’s *d*/Hedge’s *g*	
		^a^Standardized response mean	
	Other information	^a^Example of one goal or goal areas	
		^a^Use of parametric or non-parametric tests	

^a^Denotes optional, but useful information to include in GAS studies. When included the researchers should provide sufficient details on the construction (e.g. patient/caregiver/clinician interviews, literature review) and application of the inventory (e.g. were subjects able to set goals that were not on the menu)

^b^Not applicable

This systematic review has potential limitations. The initial literature screening was conducted by only one reviewer during the title/abstract screening and the full-text screening stages. This may have decreased the number of relevant studies identified for use in the systematic review. As highlighted by Rachel and colleagues [70], GAS is heterogeneous group of methodologies, and lack of GAS details included in published reports poses challenges into to a synthesis of most common GAS implementation practices.

## Conclusions

In summary, GAS with caregiver input during goal setting was utilized in a limited number of randomized controlled trials, primarily in pediatric patients and adults with dementia. A large majority of the studies did not report the specifics of how GAS was implemented, and this may compromise the ability of others to reproduce work or deploy GAS in new studies. While this systematic review has some limitations, such as having one reviewer during the identification and screening of the literature, given the significant heterogeneity in the design and implementation of GAS with caregiver input during goal setting, we believe a consensus on GAS methods and best practices, with input from both clinicians and patients, is warranted.

### Electronic supplementary material

Below is the link to the electronic supplementary material.

Supplementary File 1: Complete search strategy

Supplementary File 2: Data extraction file

Supplementary File 3: Risk of bias assessment

## Data Availability

All data generated or analyzed during this study are included in this published article.

## List of abbreviations

COPM: Canadian Occupational Performance Measure
GAS: Goal attainment scaling
SMART: Specific, Measurable, Achievable, Relevant, and Time-Bound
