# Supplementary material for: A systematic review of goal attainment scaling implementation practices by caregivers in randomized controlled trials
Source: J Patient Rep Outcomes. 2024 Mar 26;8:37. doi: 10.1186/s41687-024-00716-w (PMC10965877; doi:10.1186/s41687-024-00716-w)
Supplement: Supplementary file 1 — Complete search strategy [file 41687_2024_716_MOESM1_ESM.docx]

**Supplementary File 1: Complete Search Strategy**

**^1^Medline**

| Search terms | Articles |
| --- | --- |
| 1. (goal* adj3 (set* or plan* or attain* or achiev*)).mp. | 51893 |
| 1. (family care* or caregiver* or carer* or caretaker*).mp. | 119369 |
| 1. exp Caregivers/ | 48541 |
| 1. ((randomized controlled trial or controlled clinical trial).pt. or (Randomized or placebo or randomly or trial or groups).ab. or drug therapy.fs.) not (exp animals/ not exp humans/) | 4867941 |
| 1. exp proxy/ or prox*.mp. | 362545 |
| 1. 2 or 3 or 5 | 479777 |
| 1. 1 and 4 and 6 | 442 |
| 1. limit 8 to (english language and yr="1968 -Current") | 425 |

**Embase**

| Search terms | articles |
| --- | --- |
| 1. (goal* adj3 (set* or plan* or attain* or achiev*)).mp. | 73107 |
| 1. (family care* or caregiver* or carer* or caretaker*).mp. | 175695 |
| 1. exp Caregivers/ | 107268 |
| 1. (Randomized controlled trial/ or Controlled clinical study/ or random*.ti,ab. or randomization/ or intermethod comparison/ or placebo.ti,ab. or (compare or compared or comparison).ti. or ((evaluated or evaluate or evaluating or assessed or assess) and (compare or compared or comparing or comparison)).ab. or (open adj label).ti,ab. or ((double or single or doubly or singly) adj (blind or blinded or blindly)).ti,ab. or double blind procedure/ or parallel group*1.ti,ab. or (crossover or cross over).ti,ab. or ((assign* or match or matched or allocation) adj5 (alternate or group*1 or intervention*1 or patient*1 or subject*1 or participant*1)).ti,ab. or (assigned or allocated).ti,ab. or (controlled adj7 (study or design or trial)).ti,ab. or (volunteer or volunteers).ti,ab. or human experiment/ or trial.ti.) not (((random* adj sampl* adj7 ("cross section*" or questionnaire*1 or survey* or database*1)).ti,ab. not (comparative study/ or controlled study/ or randomi?ed controlled.ti,ab. or randomly assigned.ti,ab.)) or (Cross-sectional study/ not (randomized controlled trial/ or controlled clinical study/ or controlled study/ or randomi?ed controlled.ti,ab. or control group*1.ti,ab.)) or (((case adj control*) and random*) not randomi?ed controlled).ti,ab. or (Systematic review not (trial or study)).ti. or (nonrandom* not random*).ti,ab. or "Random field*".ti,ab. or (random cluster adj3 sampl*).ti,ab. or ((review.ab. and review.pt.) not trial.ti.) or ("we searched".ab. and (review.ti. or review.pt.)) or "update review".ab. or (databases adj4 searched).ab. or ((rat or rats or mouse or mice or swine or porcine or murine or sheep or lambs or pigs or piglets or rabbit or rabbits or cat or cats or dog or dogs or cattle or bovine or monkey or monkeys or trout or marmoset*1).ti. and animal experiment/) or (Animal experiment/ not (human experiment/ or human/))) | 5297878 |
| 1. exp proxy/ or prox*.mp. | 482553 |
| 1. 2 or 3 or 5 | 655024 |
| 1. 1 and 4 and 6 | 633 |
| 1. limit 7 to (english language and yr="1968 -Current") | 623 |

**PsycInfo**

| Search terms |  |
| --- | --- |
| 1. (goal* adj3 (set* or plan* or attain* or achiev*)).mp. | 35972 |
| 1. (family care* or caregiver* or carer* or caretaker*).mp. | 83398 |
| 1. exp Caregivers/ | 34812 |
| 1. exp Clinical Trials/ or Placebo/ or (random* or sham or placebo* or ((singl* or doubl*) adj (blind* or dumm* or mask*)) or ((tripl* or trebl*) adj (blind* or dumm* or mask*)) or (control* adj3 (study or studies or trial* or group*)) or Nonrandom* or non random* or non-random* or quasi-random* or quasirandom* or allocated or ((open label or open-label) adj5 (study or studies or trial*)) or ((equivalence or superiority or non-inferiority or noninferiority) adj3 (study or studies or trial*)) or ((pragmatic or practical) adj3 trial*) or ((quasiexperimental or quasi-experimental) adj3 (study or studies or trial*)) or (phase adj3 (III or "3") adj3 (study or studies or trial*))).ti,ab,hw. | 394717 |
| 1. exp proxy/ or prox*.mp. | 41859 |
| 1. 2 or 3 or 5 | 123770 |
| 1. 1 and 4 and 6 | 132 |
| 1. limit 7 to (english language and yr="1968-Current”) | 129 |

**Cochrane**

| Search terms |  |
| --- | --- |
| 1. (goal*NEAR/3(set* or plan* or attain* or achiev”)):ti,ab,kw | 8678 |
| 1. (family care* or caregiver* or carer* or caretaker*): ti,ab,kw | 34224 |
| 1. MeSH descriptor: [Caregivers] explode all trees | 3140 |
| 1. MeSH descriptor: [Proxy] explode all trees | 78 |
| 1. Prox*: ti,ab,kw | 17273 |
| 1. #2 OR #3 OR #4 OR #5 | 50850 |
| 1. #1 AND #6 | 964 |

**CINAHL**

| Search terms |  |
| --- | --- |
| 1. (goal* N3 (set* OR plan* OR attain* OR achiev*)) | 28980 |
| 1. (family care* OR caregiver* OR carer* OR caretaker*) | 239979 |
| 1. (MH "Caregivers") | 42287 |
| 1. (MH "Proxy") OR prox* | 56717 |
| 1. (MH (randomized controlled trials OR double‐blind studies OR single‐blind studies OR random assignment OR pretest‐posttest design OR cluster sample ) OR TI ( randomised OR randomized ) OR AB random* OR TI trial OR ( (MH (sample size) AND AB (assigned OR allocated OR control)) ) OR MH ( placebos OR crossover design OR comparative studies ) OR AB ( (control W5 group) OR (cluster W3 RCT) OR PT (randomized controlled trial)) ) NOT ( ( MH animals+ OR MH (animal studies) OR TI (animal model*) ) NOT MH (human) ) | 954392 |
| 1. #2 OR #3 OR #4 | 294288 |
| 1. #1 AND #5 AND #6 | 469 |

^1^Complete search strategy for the Medline, Embase, PsycINFO, Cochrane and CINAHL databases
