# Supplementary material for: A systematic review of goal attainment scaling implementation practices by caregivers in randomized controlled trials
Source: J Patient Rep Outcomes. 2024 Mar 26;8:37. doi: 10.1186/s41687-024-00716-w (PMC10965877; doi:10.1186/s41687-024-00716-w)
Supplement: Supplementary file 2 — Data extraction file [file 41687_2024_716_MOESM2_ESM.docx]

**Supplementary File 2: Data extraction file**

Study ID

Covidence #

Title

Country in which the study conducted

Number of study sites

Was GAS a primary or secondary outcome?

Was there any another patient-reported outcome measure (PROM) used in the study?

If yes, what was the name of the PROM?

Was the generated allocation sequence used in a sufficient manner to prevent selection bias?

Was the allocation sequence concealed in a sufficient manner to prevent selection bias?

Were the participants and personnel blinded to the intervention to prevent performance bias?

Was the outcome assessment blinded to prevent detection bias?

Was the completion rate of outcome assessment reported to prevent attrition bias?

Were all outcome assessments reported to prevent reporting bias?

Aim of study

Population description

Disorder area

Sample size of treatment group (pre-intervention)

Sample size of treatment group (post-intervention)

If follow-up, sample size of treatment group

Sample size of control group (pre-intervention)

Sample size of control group (post-intervention)

If follow-up, sample size of control group

Intervention details

Study design

Number of goals set for each subject/patient

Total number of goals set

Mean number of goals set (treatment group)

Mean number of goals set (control group)

Range of number of goals set

Were the same number of goals set for all participants/patients or different numbers of goals were set?

Who was involved in goal setting process?

Was there anyone else involved in goal setting process?

Who assessed the goal attainment?

Was there anyone else involved in assessing goal attainment?

Was there a quality assurance process included in the goal setting process?

If yes, how was the QA done?

How many levels were set for each goal?

If 8+ levels were set, how many levels were set?

Were half-point outcome levels set in this study (at either goal setting and/or follow-up)?

Which level was set for baseline?

Was a disease-specific menu used?

If yes, what was the menu's name?

Were the goals weighted?

If goals were weighted, how were they weighted?

How many times were the follow-up visits conducted?

What was the length of time between the follow-up visits?

Was there any kind of training provided to the personnel conducting the GAS?

If yes, what kind of training was provided?

Was the average time spent on setting each goal reported?

If yes, what was the average time spent on setting each goal?

Was the completion rate of GAS interview reported?

If reported, what was the completion rate of GAS interview?

Was there anything else about the goal setting interview that was unique or different in this study?

What kinds of treatment effects were discussed in the results?

Which effect sizes were reported?

Is the value of p (rho) similar to the one recommended by Kiresuk and Sherman (1968) (which is 0.3) or different?

Which statistical tests were used in the study?

If regression model or ANOVA was used, provide modeling details (e.g., what covariates were included?)

How were goal attainment ratings summarized per subject?
