# Supplementary material for: A systematic review of goal attainment scaling implementation practices by caregivers in randomized controlled trials
Source: J Patient Rep Outcomes. 2024 Mar 26;8:37. doi: 10.1186/s41687-024-00716-w (PMC10965877; doi:10.1186/s41687-024-00716-w)

**Supplementary File 3: Risk of bias assessment**

**Supplementary Figure 1.** Cochrane risk of bias tool results. D1: allocation sequence use, D2: allocation sequence concealment, D3: Blinding of the participant and personnel, D4: Blinding of outcome assessment, D5: Completion rate of outcome assessments, D6: reporting of all outcome assessments.

**
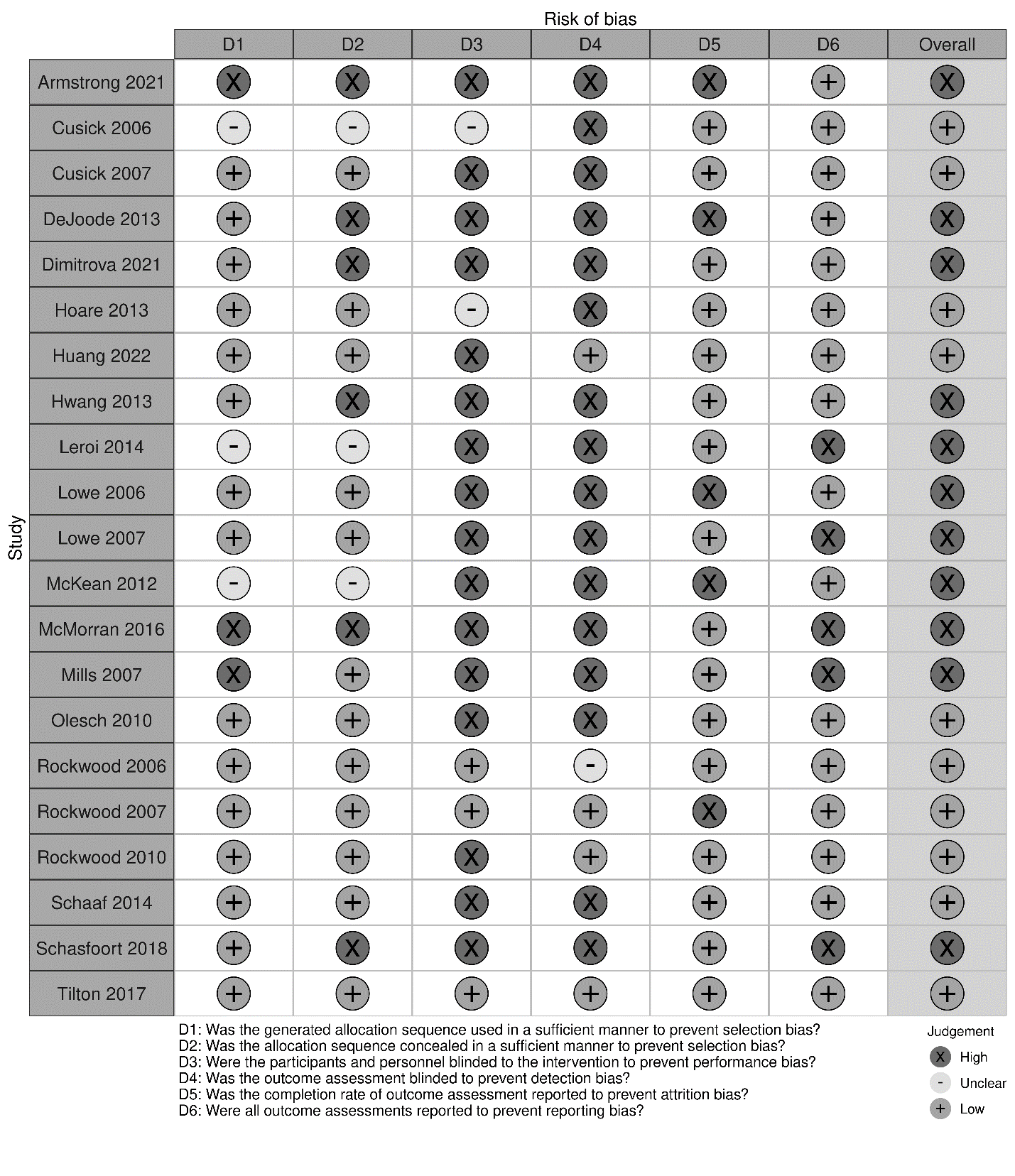
**

**Supplementary Figure 2.** Cochrane risk of bias summary results.


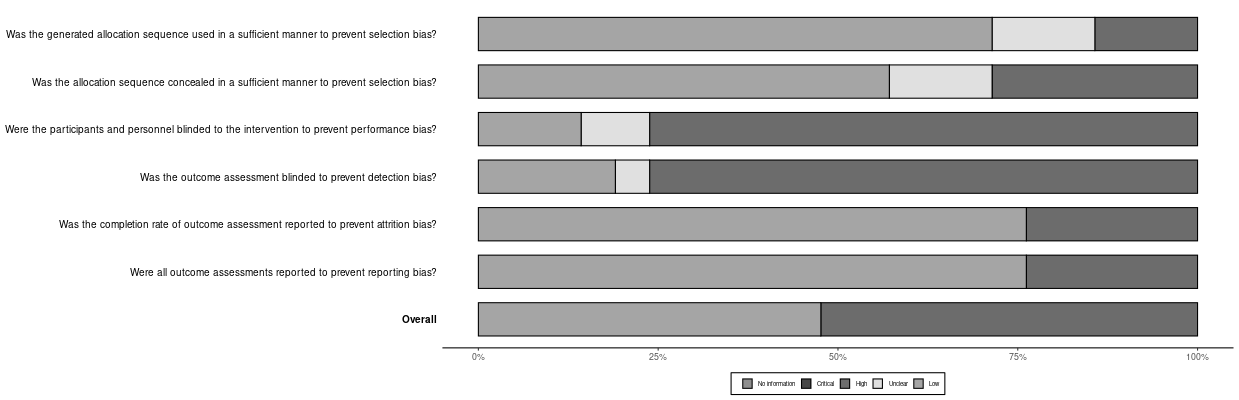

Supplement: Supplementary file 3 — Risk of bias assessment [file 41687_2024_716_MOESM3_ESM.docx]
